# An Essay on the Properties of Animal and Vegetable Life; Their Dependence on the Atmosphere, and Connexion with Each Other, in Relation to the Functions of Health and Disease

**Published:** 1846-04

**Authors:** 


### Art. XII.

*An Essay on the Properties of Animal and Vegetable life; their dependence on the Atmosphere, and Connexion with each other, in relation to the Functions of Health and Disease.* By Edward James Shearman, m.d., &c. &c.—*London,* 1845. 12mo. pp. 175.

Our readers are well aware that we have always drawn a broad line of distinction between those works addressed to the general public which tend to enlighten them with regard to the healthful regulation of their bodily functions and the avoidance of causes of disease, and those which profess to bring down the rules for the diagnosis and treatment of disease within the popular capacity. Of the former subjects they cannot, in our estimation, know too much, and every particle of fresh information has its use. In regard to the latter class, we regard the old adage "A little knowledge is a dangerous thing," as pre-eminently true; there being, in our minds, no safe medium between that entire abstinence from all interference with nature's own method of curing diseases (the access of which is due, in nine cases out of ten, to some infringement of her laws), befitting a state of entire ignorance of her operations, and that kind of interposition which is justified by extensive observation of the phenomena of disease, and full acquaintance with the most successful methods of treatment which experience can dictate.

The little volume, whose title we have quoted, fairly belongs to the first of these categories, and it has merits which distinguish it from other works of its class, in the more comprehensive survey which it takes of the general phenomena of life, and in the introduction of numerous facts derived from the most modern sources of information. But, on the other hand, we feel in duty bound to state that it is disfigured by numerous errors of a very grave description, some of which display either a positive ignorance of the elements of science, which we should have scarcely imagined possible in a well-educated physician, or a lamentable want of power of accurate expression, both of which faults are alike injurious to an author who addresses the general public, or indeed to any author whatever; whilst others result from a want of scientific discrimination, which leads to the enunciation of crude theories as admitted scientific facts. A few short extracts will serve to justify our criticism; we shall not tire the patience of our readers by much comment, nor by lengthy quotations.

"It is an established fact in natural philosophy that transparent convex lenses absorb, and opaque concave lenses reflect, the rays of light." (p. 13.)

We should like to know where Dr. Shearman learned his optics.

"The mind of man evidently depends upon the organization of the brain, and when the organs cease to act, in death, the brain, and consequently the mind, is, as will be discovered as we proceed, converted into gas, to supply the wants of vegetable matter, in the same way as the rest of the animal creation." (p. 14.)

Although "imagination may trace the noble dust of Alexander, till we find it stopping a bunghole," yet our author's powers far transcend those of the poet; for, by some undescribed process, he seems to detect the solid mind of a philosopher in the corn and potatoes of the next generation (a new relation which we recommend to the consideration of the political economists); whilst the lighter mind of the poet may find a fitter habitation amongst roses and violets, carnations and lilies; the mind of the physician, we presume, will furnish food to medicinal plants, and that of the architect or artisan will aid the development of timber-trees. Two pages further on we have the following novel theory of calorification:

"Whenever hydrogen and oxygen meet in the chemical laboratory, under the influence of galvanic action, they are decomposed, water is formed, and heat is set free. The nerves supplying the lungs sufficiently account for the galvanic battery." (p. 16.)

"Which fully accounts for the milk of the cocoa-nut," we are tempted to add by way of parallel. It would be difficult, we think, to put together anything more incongruous than the ideas contained in the above quotation. In our simplicity we had always imagined oxygen and hydrogen to be undecomposable substances; we were further taught in our younger days, that whilst an electric spark would cause these two gases to unite and form water, a galvanic current decomposed water, and resolved it into its component gases. Moreover, we are at a loss to perceive how "the nerves supplying the lungs account for the galvanic battery;" unless the author means to assert that they convey galvanic influence from the nervous centres, which no one has yet succeeded in proving, all evidence on this point being decidedly negative.

In endeavouring to explain the nature of the respiratory process, and to harmonize the rival theories of Liebig and Mulder, Dr. Shearman involves the subject in a most perplexing jumble, through which it must be difficult, if not impossible, for any uninformed reader to find his way to the simple truth, that oxygen is imbibed in the lungs in exchange for carbonic acid given off, the reverse process taking place in the tissues; and that the blood is the carrier of these gases between the pulmonary and systemic capillaries, in all cases in which the air does not come into direct relation with the latter.

In a subsequent chapter, on secretion, we are told that "the arteries, when supplied with proper nerves, have the power of secreting that peculiar fluid from the blood which the various organs require; the real cause of this principally depends upon that peculiar galvanic power which the nervous system has over the various organs." (p. 32.) Here again we find an ignorance of the tendency of recent investigations, which we should not have expected in a writer who seems to be on many other points "well up" with modern science. Everything indicates that the secreting process is no more dependent upon the nervous system in the animal than it is in the plant, that its immediate instruments are the same in both, namely *cells*, and that the *vessels* are only subservient to it, by furnishing a constant supply of the requisite material.

In the same chapter, under the head of perspiration, after learning that about thirteen ounces of watery fluid are excreted from the skin of a healthy adult in the form of insensible perspiration every twenty-four hours, and that this is loaded with nitrogen, "which is taken into the stomach with the food, in the form of nitrogen gas of the air" (of which last statement we must confess ourselves unable to comprehend the precise meaning), we meet with the following novel and startling announcement:

"But the skin has the power of excreting a large quantity, not only of fluid, but of nitrogen. Eleven grains of matter are exhaled from the skin per minute, or thirty-three ounces in twenty-four hours; and of this eighty-eight per cent. is solid,—principally nitrogen." (p. 47.)

Eighty-eight per cent. of thirty-three ounces is precisely twenty-nine ounces, a quantity greater than the average amount of solid matter daily taken in by an adult as food. Where *did* Dr. Shearman find such a statement as this? Certainly not in the works of Billing, Bird, Brongniart, Carpenter, Dumas, Johnson, Liebig, Müller, and Mulder, to whom he refers as his physiological authorities. That the above assertion is not a mere clerical error, but expresses the author's convictions, appears from a repetition of it in the appendix (p. 161), where it seems to rest upon the authority of Müller. The source of the author's mistake is evidently to be found in the idea that the analysis of the *solid* matter of the cutaneous exhalation,—which does not average above 1 or 2 per cent.,—represents that of the entire secretion, of which 98 or 99 parts are water. How any man in his sober senses could make such a mistake, we are at a loss to conceive.

We are sorry to feel called upon to express so strong an opinion as to the demerits of this treatise. Dr. Shearman may be, and we gather from many of his pages that he is, a judicious practitioner and an estimable man. But he has assuredly much mistaken his vocation, in committing himself to authorship. The general plan of the book, as we have already stated, seems to us to deserve much commendation. It is in its execution that the author has so lamentably failed; and we cannot but regret that we have felt ourselves called upon to expose his errors.

